# Live to Work or Work to Live? An Age-Moderated Mediation Model on the Simultaneous Mechanisms Prompted by Workaholism Among Healthcare Professionals

**DOI:** 10.3389/fpsyg.2019.00868

**Published:** 2019-05-07

**Authors:** Paola Dordoni, Sascha Kraus-Hoogeveen, Beatrice I. J. M. Van Der Heijden, Pascale Peters, Ilaria Setti, Elena Fiabane

**Affiliations:** ^1^Department of Brain and Behavioural Sciences, University of Pavia, Pavia, Italy; ^2^Faculty of Economics and Management, HAN University of Applied Sciences, Nijmegen, Netherlands; ^3^Institute for Management Research, Radboud University, Nijmegen, Netherlands; ^4^Schouten Global, Centre of Research, Knowledge and Innovation, Zaltbommel, Netherlands; ^5^Faculty of Management Sciences, Open University of the Netherlands, Heerlen, Netherlands; ^6^Faculty of Economics and Business Administration, Ghent University, Ghent, Belgium; ^7^Business School, Hubei University, Wuhan, China; ^8^Kingston Business School, Kingston University, London, United Kingdom; ^9^Center for Strategy, Organization and Leadership, Nyenrode Business Universiteit, Breukelen, Netherlands

**Keywords:** workaholism, perceived workload, emotional exhaustion, job satisfaction, age, healthcare professionals

## Abstract

The “aging population” implies an increased proportion of older professionals and a growing demand for healthcare services. Healthcare professionals are often highly committed to their work which can be reflected in high levels of workaholism, being a double-edged sword that can prompt both positive and negative mechanisms, differently affecting younger and older healthcare workers. The present study aims to gain insights into the relationships between healthcare professionals' age, workaholism and job satisfaction, by estimating the sequential mediating roles of workload perceptions and emotional exhaustion. We used original survey data, including information on 750 healthcare professionals. Overall, the negative relationship between workaholism and job satisfaction was shown to be sequentially (and partially) mediated by workload perceptions and emotional exhaustion. Multi-Group SEM analyses revealed differences across three age groups (under 35; between 35 and 50; over 50). Only in the two younger age groups, we found a direct and positive relationship between workaholism and job satisfaction. In all age groups, we found the negative relationship between workaholism and job satisfaction to be sequentially (and partially) mediated by workload perceptions and emotional exhaustion. The indirect effects were relatively stronger in the younger age group. Workaholism can prompt both a “gain spiral” and “a loss spiral” among healthcare professionals. The first reflects workaholism to function as a job resource fostering job satisfaction (only for the two younger age groups). The second reflects workaholism to function as a job demand reducing job satisfaction. This mechanism was shown to be stronger with an increasing age.

## Introduction

All over the world, the impact of the aging and dejuvenization of the working population on the labor market is felt strongly by healthcare organizations and their staff (cf. Camerino et al., 2006; Wang and Shi, 2014). In particular, both aging and dejuvenization have led to a growing demand for healthcare services, posing higher workloads, and emotional demands on professionals in the healthcare sector (Glomb et al., 2004; Uthaman et al., 2016; Hodgkin et al., 2017), who are aging themselves (Buchan et al., 2015). Particularly for the older category of healthcare professionals, these high job demands (see Karasek, 1979; Johnson and Hall, 1988; Alarcon, 2011; Makkai, 2018; workload, time pressure, ambiguity, conflict, stress, workload, and tension are among the most important job demands which lead to burnout) may be hard to cope with (Bakker et al., 2000; Aiken et al., 2002b; Hedge and Borman, 2012; Uthaman et al., 2016; Stankiewicz-Mróz, 2018), possibly leading to increased staff turnover and decreased retention (De Gieter et al., 2011; Sawatzky and Enns, 2012; Heinen et al., 2013; Gao et al., 2017; Ravenswood et al., 2017).

In view of the need to maintain a motivated workforce and to reduce early turnover and retirement intentions among healthcare professionals (cf. Armstrong-Stassen and Stassen, 2013), healthcare organizations may find themselves in a difficult situation (Heisler and Bandow, 2018). In fact, these organizations increasingly need to focus on ways to enhance their employees' job satisfaction, which can be defined as “*a global feeling about the job or as a related constellation of attitudes about various aspects or facets of the job”* (Lu et al., 2005, p. 212). Healthcare professionals' job satisfaction has been shown to be a core factor in achieving high quality of service delivery (Lu et al., 2005; Bratt and Gautun, 2018).

So as to protect and foster their professionals' job satisfaction, healthcare organizations need to adjust their working conditions (Stone et al., 2007; Bratt and Gautun, 2018), for example by reducing professionals' perceived workload, as this is associated with increased emotional exhaustion (cf. Deckard et al., 1994; see also Brewer and Shapard, 2004; Zito et al., 2016; Gómez-Urquiza et al., 2017; Hatch et al., 2018). Likewise, workload should be aligned with their personal characteristics (i.e., age). In particular, a large amount of previous research already investigated the impact of age on employee burnout. Some studies have found no significant correlation, while others have found such correlations [see the exemplary meta-analysis by Brewer and Shapard (2004) who concluded that there is a small negative correlation between age and emotional exhaustion]. As it appears that higher age is associated with lower burnout, it is of utmost importance to better understand the role of age in research on the relationship between workaholism and job satisfaction, and its underlying mechanisms. Especially as the association may be confounded with years of experience and survival bias (i.e., those employees who survive” early job stressors and do not quit might have much better prospects (see also Maslach and Leiter, 2016).

In addition, the literature has reported some personality traits (i.e., workaholism [cf. Guglielmi et al., 2012]) to be risk factors for job satisfaction, since these may threaten and deplete personal resources (Burke and MacDermid, 1999; Burke et al., 2006; Cunningham et al., 2008; Guglielmi et al., 2012). For example, earlier studies found that healthcare professionals reported high levels of workaholism (Burke et al., 2006; Kubota et al., 2010; Balducci et al., 2016; Nonnis et al., 2017), which can be defined as “*… the tendency to work excessively in a compulsive way”* (Schaufeli et al., 2009a, p. 250). This concept refers to the combination of *working excessively* and *working compulsively*. Workaholics typically allocate as much time to work as possible, sometimes creating even more work for themselves than is necessary, as they are obsessed with their work and unable to detach from their duties (cf. Oates, 1971; Balducci et al., 2016). Recent empirical evidence corroborates the assumption that workaholism is a personal characteristic that is associated with the individual perception of an ample amount of demanding tasks and responsibilities (i.e., high job demands) since it may give reason for the necessity to dedicate an extraordinary amount of time to work (Mazzetti et al., 2016a). Therefore, workaholism can be viewed as an important personal challenge for professionals which already has been examined in relation to job satisfaction (Burke, 1999; Burke et al., 2006; Guglielmi et al., 2012; Clark et al., 2016). Strong positive relationships were found between workaholism and job workload (Kanai and Wakabayashi, 2001), and even worse, the relationship between workaholism and emotional exhaustion (that is, the depletion of personal resources) seems to be mediated by job demands (Taris et al., 2005). It is plausible that workaholics may be motivated to work based on internal “should” (an inner driver), rather than based on an intrinsic motivation to work (Spence and Robbins, 1992), so that they may not experience true satisfaction in their work (see also Ryan and Deci, 2000; Graves et al., 2012). In other words, it seems that workaholics do not receive more rewards for their efforts (Burke, 2001; Shimazu and Schaufeli, 2009; Aziz and Moyer, 2018).

In view of the account above, we can conclude that healthcare workers' job satisfaction is typically considered as one of the key indicators of well-being (Sheward et al., 2005; Scheibe et al., 2015). Moreover, it has been shown to be a core factor in achieving high quality of service delivery (cf. Lu et al., 2012). Job satisfaction is a complex and multifaceted concept, since it depends both on the nature of the job and on the individual's expectations of what the job should provide (Liu et al., 2011; Lu et al., 2012). Some scholars have argued that personal characteristics (in this study, workaholism and age) have more impact than working conditions when it comes to explaining individual reactions to work (Cunningham et al., 2008). The existing literature has revealed both positive (Burke, 1999) and negative associations between workaholism and job satisfaction (Clark et al., 2016) and scholars have not reached a shared opinion yet (cf. Burke et al., 2006). Moreover, the role of age is not clear in this regards. Although some studies have argued that personal characteristics have more impact on individuals' job satisfaction than their working conditions (Cunningham et al., 2008) (such as workload), both types of factors may play an important role.

In order to contribute to the scholarly and societal debates on working conditions, workaholism and healthcare professionals' job satisfaction, we will build on the Conservation of Resources theory (Hobfoll, 1989) and the Life-span development theory (Carstensen, 1995) to further explore the ambiguous relationships between workaholism, workload, emotional exhaustion and job satisfaction, and the moderating role of age in these relationships. Our study aims to contribute to the theoretical and empirical debates on the relationship between workaholism and job satisfaction in two ways. First, we hope to shed more light on the underlying mechanisms that can explain the ambiguous character of this relationship as revealed in the literature. Second, we use Multi-Group Structural Equation Modeling (SEM) to test for age-moderated mediation (Dordoni, 2017).

## Theory and Hypotheses

We build on the Conservation of Resources theory (COR) (Hobfoll, 1989) which is based on the assumption that “*people strive to retain, protect, and build resources and that what is threatening to them is the potential or actual loss of these valued resources*” (Hobfoll, 1989, p. 513). The COR theory suggests that people want to protect their limited personal resources (defined as “*those objects, personal characteristics, conditions, or energies that are valued by the individual or that serve as a means for attainment of these objects, personal characteristics, conditions, or energies*” (Hobfoll, 1989, p. 516) (e.g., time, physical energy, emotional energy, attention). At the same time, individuals are highly motivated to engage in behaviors that contribute to the accumulation of additional resources for the future. The threat of a net loss of resources or the lack of resource gain following people's investments can result in psychological stress (Hobfoll, 1989). In this view, environment circumstances may threat or cause a depletion of people's resources. When losses occur, however, individuals may apply resource conservation strategies by seeking resources available in order to adapt. Those with fewer resources are more vulnerable to resource loss, whereas those with greater resources are less vulnerable to resource loss and are more capable of resource gain (Hobfoll, 2001). Therefore, initial loss begets future loss (“*loss spiral*”). Conversely, initial resource gain begets future gain (“*gain spirals*”).

Applying COR theory to our study, when individuals perceive a threat or an actual loss of resources (in our empirical work, indicated by professionals' higher workload perceptions and more emotional exhaustion, respectively), or fail to receive sufficient return on their investment of resources (i.e., emotional exhaustion in our model), they experience higher levels of distress which leads to negative work outcomes (i.e., in our model, less job satisfaction). This mechanism can be referred to as the so-called ‘loss-of-resources-spiral’ (cf. Hobfoll, 1989). Despite the negative consequences associated with workaholism, in different contexts, it is regarded as a socially acceptable behavior and even encouraged within companies across various industries (Furnham, 1997). Moreover, workaholics who are enthusiastic about their work are likely to have greater life satisfaction (Bonebright et al., 2000). Studies suggested that dispositional characteristics may represent both personal demands and resources and can play as initiators of the JD-R model processes (Van den Broeck et al., 2013; Mazzetti et al., 2016a). Additionally, Wojdylo et al. (2013) suggested that workaholic employees might be able to escape from negative emotions and feelings of adequacy. Thus, work can provide them with a sense of self-confidence and psychological safety. Following this line of reasoning, workaholism may be perceived as a personal challenge which can either function as a personal demand when associated with individuals perceiving higher job demands, or as a personal resource motivating individuals for their tasks and leading to more job satisfaction (cf. Buelens and Poelmans, 2004; Shimazu et al., 2015; Clark et al., 2016). In other words, workaholism can prompt a so-called “gain-of-resources-spiral” (cf. Hobfoll, 1989).

In fact, when workaholism is perceived as a personal challenge or as a personal resource, rather than as a personal demand (cf. Buelens and Poelmans, 2004), it is plausible that people do not perceive a threat or an actual loss of resources. In that case, workaholic professionals may not experience more workload and, in turn, they may gain from their workaholic behavior, leading to work pleasure. Therefore, we first examine both the so-called “gain of resources spiral” and the “loss of resources spiral,” the latter by testing a chain of associations wherein it is assumed that “workaholism” impacts healthcare professionals' “workload perceptions,” resulting in increased “emotional exhaustion,” eventually affecting their job satisfaction.

**Hypothesis 1**: Workaholism has a direct relationship with healthcare professionals' job satisfaction (from now on called “the gain spiral hypothesis”).
**Hypothesis 2**: The relationship between workaholism and job satisfaction is mediated by healthcare professionals' workload perceptions and emotional exhaustion (from now on called the “loss spiral hypothesis”).

Our research design enables us to investigate the moderating effect of a healthcare professional's age in the “gain spiral” (i.e., Hypothesis 1) and “loss spiral” (i.e., Hypothesis 2) hypothesized above. That is, we test whether the hypothesized direct effect (workaholism—job satisfaction) and the sequential indirect effects (workaholism—workload perceptions—emotional exhaustion—job satisfaction) affect the younger age group of healthcare professionals differently in comparison with the middle-aged and older age groups. Aligning Hobfoll's COR theory (Hobfoll, 1989, 2001) with life-span development theories (Carstensen, 1995; Higgins, 1997), we assume that younger healthcare professionals may perceive time as rather “open-ended” (Carstensen, 1995). Therefore, they may gain more personal resources, such as energy from workaholism, anticipating more skills gains and career opportunities and success (Higgins, 1997). However, following COR theory, younger healthcare professionals can also be expected to be more vulnerable to perceptions of higher workload associated with workaholism, since they have gained less personal resources, such as work experience and skills over the life-span in comparison with their older peers (cf. Froehlich et al., 2015). Therefore, we expect that both the “gain spiral” and the “loss spiral” are stronger among the younger age group in comparison with the two older age groups, and hypothesize differences across the three groups (Dordoni, 2017).

**Hypothesis 3**: The assumed relationships (see Hypotheses 1 and 2) are not similar for professionals across three distinguished age groups [under 35 (younger professionals); 35 to 50 (middle-aged professionals); and over 50 years old (older professionals)].

## Methods

### Sample and Procedure

This study takes an integrative approach, including both individual and organizational factors to explain the role of workaholism in healthcare professionals' job satisfaction. The data for this study (*N* = 750) was collected by means of a stratified sample from a hospital in North Italy that can be considered representative for the composition of professionals in the healthcare sector. After approval of the ethics committee (University of Pavia), written informed consent was obtained from the participants and a self-administered questionnaire was distributed among four groups of healthcare professionals (i.e., nurse aides, nurses, physicians, and physiotherapists). Participants filled out a survey and posted it in a private box. Completing the survey took the respondents, on average, 15 min. In order to guarantee anonymity, code numbers were placed on the completed survey after they were returned to the researchers.

The majority of the respondents were females (74%) and 12% were under 35 years old; 56% were 35 to 50 years old; and 30% were over 50 years old (2% missing cases for respondent's age). As regards the different professional categories, 11% of the respondents were employed as nurse aides; 57% were nurses; 16% were physicians; and 12% were physiotherapists (4% missing cases for professional category). Moreover, 23% of the respondents were employed in the hospital for 1 to 10 years; 24% for 11 to 20 years; and 52% for over 20 years (1% missing cases for tenure). 13% held part-time jobs, while 87% held full-time jobs. With regard to type of shift, 32% did not work in shifts; 16% only worked in day shifts; and 50% also worked in night-time shifts (with 2% missing cases for type of shift).

### Measurements

Three latent variables, using Likert scales' answering categories, were included in the survey. Factor structures and scale reliabilities were tested using Confirmatory Factor Analysis (CFA) and Cronbach's alphas, respectively.

*Workaholism* was measured with two subscales from the shortened version of the ten-items *Dutch Work Addiction Scale* [DUWAS; Schaufeli et al., 2009b] [i.e., “work excessively” 5 items (Cronbach's alpha = 0.86), and “work compulsively” 5 items (Cronbach's alpha = 0.93)]. A four-point rating scale was used, ranging from 1 (= almost never) to 4 (= almost always). The standardized factor loadings for “work excessively” ranged from 0.47 to 0.60, and for “work compulsively” from 0.45 to 0.76, with all items having significant loadings on the intended factor. For the subsequent SEM analysis, the two subscales were combined in order to create a second-order construct (Schaufeli et al., 2009b), that is, workaholism (Cronbach's alpha = 0.94). The factor loadings of both subscales on this second-order construct were 0.98 and 0.79, for “work excessively” and “work compulsively”, respectively, and both factors appeared to have a significant contribution to the second-order construct.

*Workload Perceptions* was measured by means of a six-item scale (*Workload*; Leiter and Maslach, 2000) that was previously validated for Italian language by (Borgogni et al., 2005). A five-point rating scale was used, ranging from 1 (= strongly disagree) to 5 (= strongly agree). Cronbach's alpha was 0.79 and the standardized factor loadings ranged from 0.33 to 0.79, with each item having a significant contribution to the latent construct.

*Emotional exhaustion* was measured by means of a five-item scale (Schaufeli et al., 1996; Borgogni et al., 2005; MBI-GS; Maslach Burnout Inventory-General Survey) using a seven-point rating scale ranging from 1 (= never) to 7 (= daily) (Cronbach's alpha = 0.75). The standardized factor loadings ranged from 0.75 to 0.87, with each item having a significant contribution to the latent construct. This scale has been widely used in healthcare research settings (Leiter and Maslach, 2009; e.g., Lasalvia et al., 2009; Fiabane et al., 2013).

*Job satisfaction* was measured with the widely-used one-item scale from Aiken et al. (2002a), with answering categories ranging from 1 (= very unsatisfied) to 4 (= very satisfied).

*Moderator. Age* was categorized into three groups. The first group included those healthcare professionals being under 35 years old (*N* = 82); the second group included those aged 35 to 50 years old (*N* = 397); and the third group comprised those aged over 50 years old (*N* = 212) [see Van der Heijden (2001) for a justification for this age categorization)].

*Control variables*. We controlled for some key characteristics of healthcare professionals that were shown to be predictive for workload perceptions, emotional exhaustion, and job satisfaction, in previous scholarly work. Specifically, we controlled for *gender* (with reference category “male”); *professional category* (with reference category “physicians”) (Fiabane et al., 2012); *part-time* (with reference category “not part-time”); and *shiftwork* (with reference category “having no shiftwork”) (Burke et al., 2006).

### Analyses

All analyses were performed using Mplus software (Muthèn and Muthèn, 2012). First, we investigated the discriminant validity of our constructs by testing the measurement model, using CFA. Second, we conducted preliminarily analyses to explore the relationships in our model. By conducting SEM analyses, we tested the hypothesized relationships in our research model. We compared the fit of the hypothesized mediation model to several direct effects' models, using the full sample. The goodness of fit was evaluated based on the χ^2^ goodness-of-fit statistic and some alternative fit indices. Specifically, the Comparative Fit Index (CFI) was computed, considering values over 0.90 to be a good fit. For the Root Mean Square Error of Approximation (RMSEA), values up to 0.08 were viewed to represent a reasonable model fit. Finally, the Standardized Root Mean Square Residual (SRMSR) was computed, considering values under 0.09 to indicate a good model fit (Hu and Bentler, 1999).

Based on the outcomes of the direct effects' models, we selected the best fitting model in order to further examine its invariance across the three age groups by using Multi-Group SEM analysis.

## Results

### Preliminary Analyses

Table 1 presents bivariate correlations for all model variables and portrays that workaholism was significantly correlated with both workload perceptions and emotional exhaustion. Furthermore, workload perceptions had a significant correlation with emotional exhaustion and job satisfaction. Emotional exhaustion was significantly correlated with job satisfaction. Strikingly, workaholism was not significantly correlated with job satisfaction, which may be explained by the multiple underlying mechanisms (e.g., the “loss spiral” and the “gain spiral”) that have their effects in opposite directions (cf. Burke et al., 2006; Guglielmi et al., 2012). Moreover, this outcome might also be explained by the assumed differences in age groups. Based on the results of our preliminary analyses, we concluded that it is meaningful to test the mediation paths running from workaholism to job satisfaction via workload perceptions and emotional exhaustion (cf. Baron and Kenny, 1986).

**Table 1:** Correlation matrix (*N* = 750).

**Variable**	**1**	**2**	**3**	**4**	**5**	**6**	**7**	**8**	**9**	**10**	**11**	**12**	**13**	**14**	**15**
1. Workaholism	−														
2. Workload perceptions	0.77^***^	−													
3. Emotional exhaustion	0.57^***^	0.78^***^	−												
4. Job satisfaction	−0.04	−0.21^***^	−0.36^***^	−											
5. Males^a^	0.12*	0.16^**^	0.16^**^	−0.00	−										
6. Nurse aides^a^	0.04	0.02	0.04	−0.01	0.06	−									
7. Nurses^a^	0.03	0.11*	0.18^***^	−0.01	0.32^***^	−0.88^***^	−								
8. Physicians^a^	0.08	0.03	−0.17^**^	0.03	−0.50^***^	−0.56^**^	−0.93^**^	−							
9. Physiotherapists^a^	−0.18^**^	−0.27^***^	−0.16^**^	−0.00	0.01	−0.51^**^	−0.88^***^	−0.59^**^	−						
10. Age	0.00	−0.01	−0.00	−0.02	−0.03	0.20^**^	−0.28	0.23	0.06	−					
11. Tenure	0.01	−0.02	0.02	0.02	0.21^***^	0.03	0.09	−0.18^**^	0.04	0.53^***^	−				
12. Part-time work^a^	0.07	0.11	0.05	0.03	−0.61^***^	0.22	−0.23^**^	0.33^***^	−0.07	−0.18^**^	−0.23^***^	−			
13. No shiftwork^a^	0.07	0.04	0.04	−0.03	0.27^***^	−0.21^**^	−0.03	−0.06	0.28^***^	0.27^***^	−0.07	−0.38^***^	–		
14. Only daily shiftwork^a^	0.01	−0.03	0.04	0.01	0.10	0.13	0.02	−0.32^***^	0.14	0.09	−0.01	−0.11	−0.79^***^	–	
15. Also night–time shiftwork^a^	−0.07	−0.02	−0.06	0.02	−0.30^***^	0.10	0.01	0.22^**^	−0.35^***^	0.29^***^	0.07	0.45^***^	−0.97^***^	−0.89^***^	−

*** p < 0.001;

** *p < 0.01*.

a *1 = yes, 2 = no*.

### Testing the Measurement Model

We estimated a measurement model including job satisfaction and the latent constructs for workaholism, workload perceptions and emotional exhaustion. Using SEM analyses, and including both CFA and path modeling, we modeled these four study variables as discussed under Methods. Moreover, we tested the hypothesized mediation model incorporating the two distinguished sequential mediators. In comparison with the other models that were tested (the direct effects' model and the model using single mediation variables), the sequential model appeared to have the best fit with the data (see Table 2, Model G). Therefore, the sequential model was also used in the subsequent Multi-Group SEM aimed to test for a possible moderation effect of age. The CFI fit indices of all tested models appeared to be below the recommended cut-off point of 0.90 for a good fit. As the CFI depends on the average size of the correlations in the model (Kenny, 2015), the low CFI fit indices can be explained. Due to the high number of control variables (and dummy variables) in our analyses, which have low correlations with multiple variables, the average correlation is low (see Table 1). This has a negative impact on the CFI fit indices. However, both the RMSEA (acceptable fit below 0.08), and the SRMSR (acceptable fit below 0.09), indicated an acceptable model fit (cf. Hu and Bentler, 1999).

**Table 2:** Goodness of fit indices for the distinguished models (*N* = 691).

		**Chi-Square Test of Model Fit**	**DF**	**Model Comparison (Δ Chi-Square)**	**Δ DF**	**RMSEA**	**CFI**	**SRMR**
A	Workaholism—job satisfaction	1564.325	404			0.064	0.809	0.061
B	Workload perc—job satisfaction	1530.274^***^	404	34.051^**^ (A-B)	0^a^	0.064	0.815	0.059
C	Emotional exhaustion—job satisfaction	1473.563^***^	404	56.711^***^ (B-C)	0^a^	0.062	0.824	0.058
D	Workaholism—emotional exhaustion—job satisfaction	1508.969^***^	393	−35.406^**^ (C-D)	11	0.064	0.817	0.058
E	Workaholism—workload perc.—job satisfaction	1606.099^***^	404	−132.536^***^(C-E)	0^a^	0.066	0.802	0.062
F	Workload perc.— emotional exhaustion—job satisfaction	1441.822^***^	393	31.741^**^ (C-F)	11	0.062	0.828	0.056
G	Workaholism—workload perc.—emotional exhaustion—job satisfaction	1425.816^***^	391	16.006^**^ (F-G)	2	0.062	0.830	0.055

a *In case the df difference is 0, the Chi-Square comparison was tested with a df of 1*.

*** p < 0.001;

** *p < 0.01*.

Based on the sequential mediation model of the full dataset (Model G), we found two significant paths. In line with our “gain-spiral hypothesis” (i.e,. Hypothesis 1), we found a significant direct and positive relationship between workaholism and job satisfaction (β = 0.303; *p* < 0.001). In line with our “loss-spiral hypothesis” (i.e., Hypothesis 2), we found a significant indirect and negative relationship between workaholism and job satisfaction, mediated via workload perceptions and emotional exhaustion (β = −0.320; *p* < 0.001) (see **Table 4**, section A).

### Testing for Measurement Invariance

In order to test for age moderation, we compared the structural models, which were based on the scores for their latent constructs, for the three distinguished age groups separately (youngsters, middle-aged, and older healthcare professionals, respectively). In order to be able to compare the results, measurement invariance between the age groups should be present. The measurement invariance of the sequential mediation model (Model G) across the three distinguished age groups was studied using a stepwise procedure (Van de Schoot et al., 2012). The first step of this analysis comprised testing a free model (Model 1) in which all parameters were unrestricted. In a second step, we compared the fit of Model 1, respectively, with: a model in which the factor loadings were constrained to be equal (Model 2); a model in which both factor loadings and intercepts were constrained to be equal (Model 3); a model in which the factor loadings, intercepts and the errors were constrained to be equal (Model 4); and, finally, a model in which the factor loadings, intercepts, errors and the structure were constrained to be equal (Model 5) (cf. Van de Schoot et al., 2012). Our results showed that there was metric invariance. Since Model 4 did not fit significantly worse than the previous models, this model was accepted as the best model (cf. Van de Schoot et al., 2012). This implies that the meaning of the constructs, based on both the factor loadings, the intercepts and the errors, can be regarded to be equal across the distinguished age groups. The presence of metric invariance justified to compare the scores on the latent constructs across the three age groups (Van de Schoot et al., 2012). In addition, the analysis also indicated that structural invariance was absent. Indeed, the model with the fixed structure (Model 5) had a significantly worse fit (as the Chi-Square raised significantly) in comparison with Model 4 (see Table 3). Both outcomes justified the use of a Multi-Group SEM analysis for the distinguished structural paths. Again, the CFI fit indices of all the model tests appeared to be below the recommended cut-off point of.90 for a good fit. However, both the RMSEA (acceptable fit below.08), and the SRMSR (acceptable fit below.09), indicated an acceptable model fit (cf. Hu and Bentler, 1999).

**Table 3:** Measurement fit for the distinguished invariance models (N = 702).

		**Chi-Square test of model fit**	**DF**	**Model comparison (Δ Chi-Square)**	**Δ DF**	**RMSEA**	**CFI**	**SRMR**
1	Free Model	3130.298	1003			0.096	0.660	0.206
2	Loadings Fixed	2173.583^***^	990	965.715^***^ (1-2)	13	0.072	0.811	0.076
3	Loadings Intercepts Fixed	2167.722^***^	988	5.861 (2-3)	2	0.072	0.811	0.073
4	Loadings Intercepts Errors Fixed	2214.304^***^	1038	46.582 (3-4)	50	0.070	0.812	0.077
5	Loadings Intercepts Errors Structure Fixed	2288.097^***^	1092	73.793^*^ (4-5)	54	0.069	0.809	0.082

*** p < 0.001;

* *p < 0.05*.

### Testing for Age-Moderated Sequential Mediation

In Hypothesis 1 and 2, we hypothesized a direct relationship and an indirect relationship between workaholism and job satisfaction. Moreover, in Hypothesis 3, we hypothesized these relationships to be moderated by age. In other words, we expected the assumed relationships (see Hypotheses 1 and 2) not to be similar for professionals across the three distinguished age groups.

**Figure 1:**
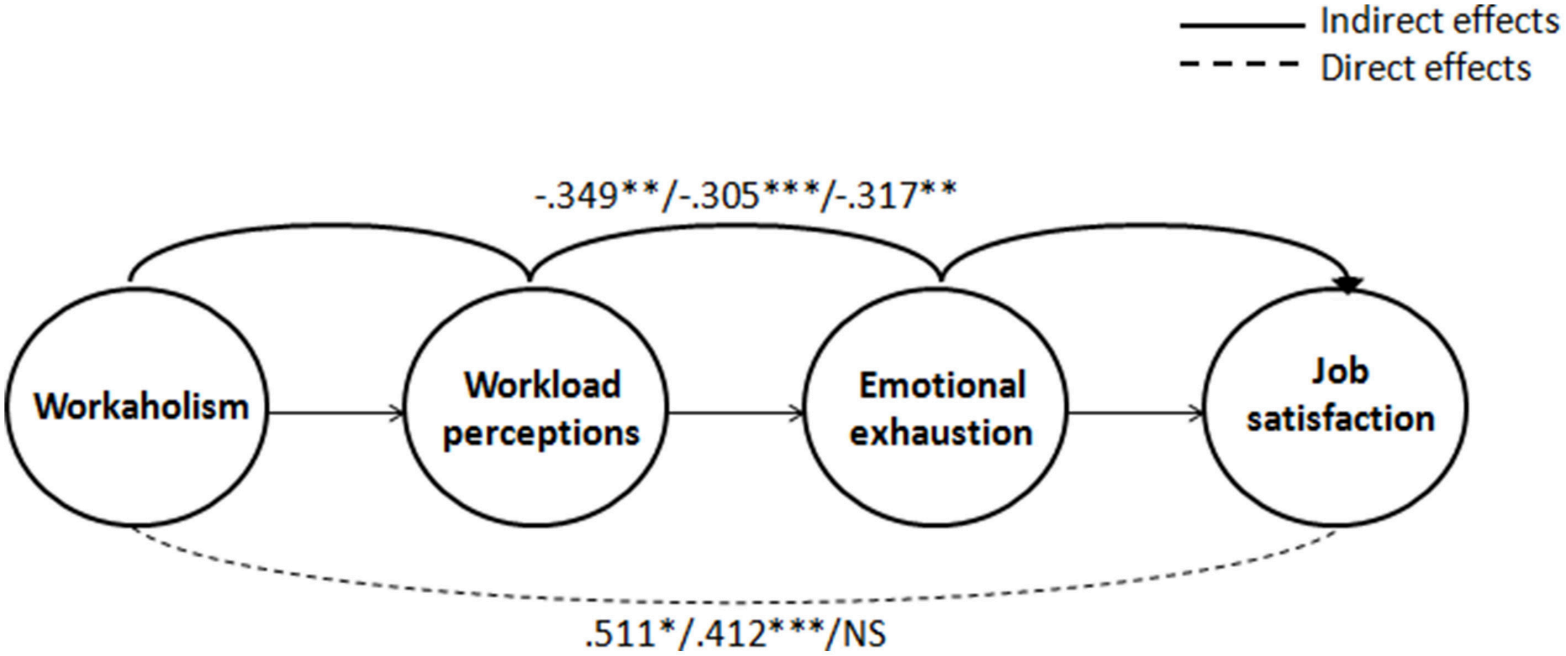
Parameter estimates for the Multi-Group SEM model (standardized coefficients). The coefficients of the different age groups are presented in the following order: Under 35 years old; 35–50 years old; and Over 50 years old). ***p < 0.001; **p < 0.01; *p < 0.05.

In line with Hypothesis 3, the Multi-Group SEM model revealed different paths across the three age groups (see Table 4, section B, and Figure 1). On the one hand, our Multi-Group SEM analysis revealed a direct and positive relationship between healthcare professionals' workaholism and job satisfaction, for the younger age group (β = 0.511, *p* = 0.026) and (to a lesser degree) for the middle-aged group (β = 0.412, *p* < 0.001), but not for the oldest age group. This confirms our expectation that younger healthcare professionals would experience the potential “gain spiral” prompted by workaholism more than their older peers. On the other hand, our multi-group SEM analysis revealed workaholism to be indirectly and negatively related to job satisfaction, sequentially mediated via workload perceptions and emotional exhaustion. Although this held true in all three age groups, we found that this negative indirect effect was stronger among the younger age group (β = −0.349, *p* = 0.007) in comparison with the middle-aged (β = −0.305, *p* < 0.001) and the older age group (β = −0.317, *p* = 0.004). This finding supports our expectation that younger healthcare professionals will also be more vulnerable to the “loss spiral” prompted by workaholism.

**Table 4:** Structural paths' results of the distinguished samples.

	**Section A**	**Section B**		
**Variables**	**Full data**	**Under 35 years old**	**Between 35 and 50 years old**	**Over 50 years old**
	**Job Satisfaction ON**			
	**β (SE)**	**β (SE)**	**β (SE)**	**β (SE)**
**DIRECT EFFECTS**				
Workaholism	0.303^***^(0.082)	0.511^*^(0.225)	0.412^***^(0.105)	0.115(0.145)
Workload perceptions	−0.061(0.110)	−0.080(0.291)	−0.182(0.141)	0.105(0.223)
Emotional exhaustion	−0.524^***^(0.070)	−0.775^***^(0.211)	−0.503^***^(0.089)	−0.486^**^(0.149)
Gender Males^b^	0.063(088)	−0.169(0.207)	0.153(0.118)	0.023(0.183)
Nurse aides^a^	0.055(142)	0.142(0.578)	−0.061(201)	0.164(0.237)
Nurses^a^	0.078(0.109)	0.593(0.359)	0.013(0.153)	0.030(0.193)
Physiotherapists^a^	0.018(0.139)	−0.036(0.363)	−0.019(0.204)	0.029(0.266)
Part–time^b^	0.078(0.112)	2.803^***^(0.744)	−0.044(0.132)	0.227(0.229)
Only day shift work^c^	0.071(0.110)	−0.176(0.423)	0.165(0.144)	−0.112(0.197)
Also night–time shift work^c^	0.019(0.087)	−0.005(0.347)	0.037(0.113)	−0.043(155)
**INDIRECT EFFECTS**				
Workaholism on job satisfaction mediated by workload perceptions and emotional exhaustion	−0.320^***^(0.056)	−0.349^**^(0.130)	−0.305^***^(0.065)	−0.317^**^(0.109)
Workload perceptions on job satisfaction mediated by emotional exhaustion	−0.421^***^(0.067)	−0.473^**^(0.168)	−0.414^***^(0.081)	−0.425^**^(0.140)
Gender on job satisfaction mediated by workload perceptions and emotional exhaustion	–	ns^d^	ns^d^	−0.203^*^(0.091)
Nurse aides on job satisfaction mediated by emotional exhaustion	-	ns^d^	ns^d^	−0.230^*^(0.114)
Nurses on job satisfaction mediated by workload perceptions and emotional exhaustion	-	−0.409^*^(189)	ns^d^	ns^d^
Nurses on job satisfaction mediated by emotional exhaustion	-	ns^d^	ns^d^	−0.220^*^(0.097)
Physiotherapists on job satisfaction mediated by emotional exhaustion	-	ns^d^	ns^d^	−0.348^*^(0.140)
R^2^	0.216^***^(0.033)	0.467^***^(0.114)	0.275^***^(0.048)	0.137^**^(0.050)

*** p < 0.001;

** p < 0.01;

* *p < 0.05*.

a *Reference category “the physicians”*.

b *1 = yes, 2 = no*.

c *Reference category “no shift work”*.

d *not significant*.

## Discussion and Conclusion

Workaholism can be viewed to be an important personal “challenge” (Buelens and Poelmans, 2004) for healthcare professionals as it can both function as a resource and a demand. With regard to the former, workaholism has the potential to energize workers and hence can create pleasure in the work of healthcare professionals, leading to more job satisfaction, possibly buffering negative emotions and affects (see Hypothesis 1). With regard to the latter, workaholism can enhance perceptions of workload and emotional exhaustion which can be experienced in lower levels of job satisfaction (Clark et al., 2016). Healthcare professionals who experience higher levels of workaholism may more frequently fall into a so-called “loss spiral” due to them being exposed to a depletion of job resources, leading to a health impairment psychological process (Del Líbano et al., 2012; Caesens et al., 2014; Clark et al., 2016) and causing feelings of emotional exhaustion (Hobfoll, 1989). This, in turn, has the potential to decrease their job satisfaction (Clark et al., 2016) (see Hypothesis 2).

Following the mechanism of the “loss spiral” (Hobfoll, 1989), professionals with fewer resources might be less capable to withstand further threats to resource losses, and consequently, being less capable of (re)gaining helpful resources as well (cf. Gorgievski and Hobfoll, 2008; Lorente Prieto et al., 2008; Xanthopoulou et al., 2009; Guglielmi et al., 2012; and the interactional perspective as used by Pervin, 1989). This may result in even more detrimental “loss cycles” or “dog-eat-dog” mechanisms.

### Differences in the Gain and Loss Spirals Between Workaholism Across Age Groups

Comparing the “gain spiral” and the “loss spiral” mechanisms across the three distinguished age groups, our findings regarding the direct effects' outcomes showed that only the younger and middle-aged groups experienced to gain job satisfaction from workaholism (see Hypothesis 3).

Aligning Hobfoll's COR theory (Hobfoll, 1989) with life-span development theories (Carstensen, 1995; Higgins, 1997), younger professionals may perceive time as rather “open-ended” (Carstensen, 1995) and may be more motivated by growth and knowledge-related goals than older professionals. This motivation can be particularly useful to achieve successful objectives, such as further personal resource accumulation, reflecting in skill development and experience which can contribute to their career development (ibid.). Younger professionals may therefore also gain more work-related pleasure through workaholism compared with their older peers, because the first category is more career-motivated and promotion-focused (i.e., more interested in growth and development) (Higgins, 1997). The older age group, in contrast, did not experience to gain any job satisfaction from workaholism. Possibly, older professionals' workaholism may foster less joy and energy as, in view of their age, they may possess more skills and experience. On the other hand, they may possess less personal resources (cf. Baltes et al., 1999), such as stamina and work capacities, and need more time to recover from work (cf. Mohren et al., 2010). This may imply that workaholism does not lead to more energy, and hence, work satisfaction.

Whereas, the direct and positive effect of workaholism on job satisfaction (the “gain spiral” prompted by workaholism) was not present among professionals who are in the final stage of their careers, we found all age groups to be vulnerable to the indirect and negative effects of workaholism on job satisfaction (the “loss spiral” mechanism prompted by workaholism). Particularly healthcare professionals in the younger age group appeared to suffer from this “loss spiral” mechanism. Possibly, the “open time horizon” of young healthcare professionals suggested by life-span development theories can provide a fruitful theoretical explanation, as these younger professionals still have to establish their careers as healthcare professionals, and have to build skills and experience in their jobs in ever more demanding workplaces. Obviously, the older groups are less vulnerable to the loss spiral. Following COR theory, this can be explained by the fact that they have already accumulated personal resources over their professional careers (skills, work experience, self-efficacy) which reduces the risk of resource depletion.

Altogether, we found evidence for both the gain and the loss spirals between workaholism and job satisfaction. In line with expectations based on our theoretical lens, younger professionals are more subject to both the gain and the loss spiral. In view of their early career stage, they are more motivated to gain personal resources, but are also more vulnerable to losing them. Older professionals might have accumulated sufficient resources. On the one hand, this may reduce their motivation to gain more resources. On the other, it also limits their vulnerability for losing resources. In view of future developments, also in the health care domain, such as enhanced workload and the need to develop new skills due to ongoing automation all age groups might find it increasingly difficult to balance the gain and loss spiral mechanisms.

### Limitations of Our Study

Even though the age-moderated mediation model allowed us to gain more insights into the positive and negative spiral mechanisms, the data was cross-sectional. As we were not able to prove the causality of the relationships studied, our results have to be interpreted with caution. Moreover, all variables were measured by means of self-reports and, therefore, common-method bias may exist (Podsakoff et al., 2003). However, a recent study showed that “in contrast to conventional wisdom, common method effects do not appear to be so large as to pose a serious threat to organizational research” (Lance et al., 2010, p. 450). In addition, this empirical study relied on a single-item measurement of job satisfaction. Even though some scholars argue and have found empirical evidence for the reliability of single-item measurements (Wanous et al., 1997), in the light of construct validity issues, future research using more elaborate measures for job satisfaction is called for. All in all, we recommend future longitudinal research, preferably using multi-wave designs allowing to test for causal relationships. Moreover, the results of this study should be cross-validated by means of additional empirical scholarly work, herewith incorporating different countries and occupational settings as well. Our findings showed that the amount of explained variance in job satisfaction decreases with age. It is plausible that the explanatory variables included in our model are more relevant for the younger age groups than for the older one. Future research could investigate some other possible personal and job-related antecedents of job satisfaction across age. In addition, possible moderator variables, such as achievement motivation, need for autonomy, to mention but a few, could be taken into account in future scholarly work in order to better understand whether the relationship between workaholism and job satisfaction can be buffered or, even further strengthened.

### Implications

Our study has important implications for both the existing literature and for managers and stakeholders in healthcare organizations. In order to enhance healthcare professionals' satisfaction, both their personal resources and job-related demands need to be considered in relation to one another. The outcomes of the current empirical work suggest that negative effects (e.g., a reduction of job satisfaction) resulting from workaholism are most likely to occur when workers' personal characteristics interact in a certain way with job-related factors (i.e., resulting in a too high workload level). Given the very limited opportunity to influence workers' personal characteristics (in this case workaholism traits), it might be more worthwhile for healthcare organizations to stimulate a work environment that does neither enhance nor reward workaholism. Empirical results from the scholarly work by Mazzetti et al. (2016b) suggests that workaholic employees are mainly motivated by an introjected regulation, but they are also prompted by external factors, such as an organizational climate that expects employees to perform overwork and, at the same time, assign inadequate rewards for these extra work efforts. In a context where organizations often encourage workaholic behavior (cf. Burke, 2001; Johnstone and Johnston, 2005; such as incentive systems promoting to work longer), interventions in this sense are particularly important (Liang and Chu, 2009; Schaufeli, 2016). Indeed, in case a workplace culture is characterized by high workload and job demands, policies and practices should be adopted in order to limit their staff to overwork in too many occasions, herewith discouraging workaholism behavior. For instance, organizations could stimulate a growth culture (that is an organizational culture that provides opportunities for personal growth; Buelens and Poelmans, 2004) rather than a pressurizing one, and should decrease job demands (i.e., workload) where necessary. Similarly, a competitive climate also positively correlates with workaholism (Keller et al., 2016). Even though the “gain and loss spirals” are two opposing mechanisms that can operate simultaneously, in practice, the positive direct effect of workaholism on job satisfaction may be overshadowed by the indirect effect of workaholism being associated with perceptions of higher workload and more emotional exhaustion. This can cause younger healthcare professionals to leave the healthcare sector, particularly when they do not have the necessary resources to cope with balancing these positive and negative mechanisms, herewith leading to the remaining older workers to suffer even more. In fact, our results demonstrate that the *process* of aging should be a priority in HRM policies and practices; the latter have to promote employees' job satisfaction throughout their entire career. In detail, our study shows that older workers respond differently to the “negative spiral” that may be caused by workaholism in comparison with younger workers, and, as such, this “negative spiral” is particularly dangerous in later career stages. In the light of the increasing portion of older workers at the labor market, our study indicates that healthcare organizations should invest in supporting their workers' sustainable employability (Van der Heijden and De Vos, 2015) and by arranging sound and facilitating “active aging” working conditions (Kooij et al., 2010; Veth et al., 2015). Workaholism has the potential to negatively affect the older age group of healthcare professionals, particularly since they lack the direct positive effect of being motivated by workaholism, in contrast with the two other age groups whose longer “time horizon” still motives them to invest in their professional careers.

In order to enhance job satisfaction among all healthcare professionals over their full careers and to prevent them from leaving the healthcare sector, both personal resources and job- and organization-related factors need to be considered by all healthcare sector stakeholders involved. Given the very limited opportunities to influence professionals' personal characteristics, it might be more worthwhile for managers in the healthcare sector to stimulate a work environment wherein HR policies and practices are adopted that limit employees to overwork structurally [cf. (Burke, 2001)] or to provide them with job resources to balance job demands. The outcomes of this study are important for both scholars and practitioners in healthcare settings. We suggest that relevant stakeholders in healthcare organizations discuss the factors that are taken into account in this study, in order to increase the transparency of perceptions regarding job demands and personal characteristics that might be associated with workaholism. After all, protecting the sustainable careers of employees throughout the life-span is a dual responsibility wherein both employers (line managers) and employees should be aware of and should prevent possible hindrances that might endanger the individual workers employability (Van der Heijden and De Vos, 2015). For manager to use the evidence-based knowledge in practice, they have to participate in leadership programs that support sustainable and health-promoting leadership, i.e., leadership that takes into account that factors at different work system level can jointly be accountable for health outcomes. In order to manage these interactions across work system levels, managers need to learn how to use dialog to address these (Dellve and Eriksson, 2017). To conclude, our results demonstrate that the process of aging should be a priority for HR policies and practices in healthcare. In the light of the increasing proportion of older professionals in the labor market, healthcare organizations should support active aging and sustainable employability across the life-span by facilitating sound working conditions (Veth et al., 2015).

## Author Contributions

All authors listed have made a substantial, direct and intellectual contribution to the work, and approved it for publication. PD, SK-H, BV, and PP worked on Design, Modeling, Analyses, and Writing. IS and EF contributed in collecting data and proofreading.

### Conflict of Interest Statement

The authors declare that the research was conducted in the absence of any commercial or financial relationships that could be construed as a potential conflict of interest.
